# Comparison of CPU and GPU bayesian estimates of fibre orientations from diffusion MRI

**DOI:** 10.1371/journal.pone.0252736

**Published:** 2022-04-21

**Authors:** Danny H. C. Kim, Lynne J. Williams, Moises Hernandez-Fernandez, Bruce H. Bjornson

**Affiliations:** 1 Brain Mapping, Neuroinformatics and Neurotechnology Laboratory, BC Children’s Hospital, Vancouver, British Columbia, Canada; 2 BC Children’s Hospital MRI Research Facility, Vancouver, British Columbia, Canada; 3 Wellcome Centre for Integrative Neuroimaging (WIN)—Centre for Functional Magnetic Resonance Imaging of the Brain (FMRIB), University of Oxford, United Kingdom; 4 Division of Neurology, Department of Pediatrics, Faculty of Medicine, University of British Columbia, Vancouver, British Columbia, Canada; University at Buffalo, UNITED STATES

## Abstract

**Background:**

The correct estimation of fibre orientations is a crucial step for reconstructing human brain tracts. Bayesian Estimation of Diffusion Parameters Obtained using Sampling Techniques (*bedpostx*) is able to estimate several fibre orientations and their diffusion parameters per voxel using Markov Chain Monte Carlo (MCMC) in a whole brain diffusion MRI data, and it is capable of running on GPUs, achieving speed-up of over 100 times compared to CPUs. However, few studies have looked at whether the results from the CPU and GPU algorithms differ. In this study, we compared CPU and GPU *bedpostx* outputs by running multiple trials of both algorithms on the same whole brain diffusion data and compared each distribution of output using Kolmogorov-Smirnov tests.

**Results:**

We show that distributions of fibre fraction parameters and principal diffusion direction angles from *bedpostx* and *bedpostx_gpu* display few statistically significant differences in shape and are localized sparsely throughout the whole brain. Average output differences are small in magnitude compared to underlying uncertainty.

**Conclusions:**

Despite small amount of differences in output between CPU and GPU *bedpostx* algorithms, results are comparable given the difference in operation order and library usage between CPU and GPU *bedpostx*.

## Background

Recent human brain MRI data sizes are continuing to increase as large imaging data repositories curate structural, functional and diffusion data with higher spatial resolution, faster temporal sampling, and higher angular [1–3]. Performing image analysis on such dataset is computationally expensive, even with clusters of CPUs working simultaneously on a single dataset [4]. By contrast, graphics processing units (GPUs) have a massively parallel structure designed with hundreds of smaller cores optimized to exploit the data level parallelism of certain applications, utilizing simpler instruction sets and distributing them over multiple cores [5, 6]. This parallelization can accelerate computationally slow processes such as data visualization, stochastic iteration, and Bayesian simulations including probabilistic tractography [2, 4–10]. A popular tool in estimating diffusion parameters for whole brain diffusion MRI is available to be run on both CPU or GPU, with GPU algorithm achieving over 100 times speed-up compared to its CPU algorithm [6, 11, 12]. Despite the GPU’s advantages in acceleration, few studies have examined whether there are differences in computational output from the CPU and GPU. In general, checking for output convergence between CPU and GPU results is important for several reasons. First, CPU and GPU both have double-precision capabilities in their compilation and runtime libraries, but the optimization of performance and speed-up of GPU binaries may restrict them to using single-precision libraries which can cause results to be different due to float-point precision differences [13, 14]. Secondly, there are differences in the CPU and GPU random number generators and operation orders in implementing Markov Chain Monte Carlo (MCMC) [15–17]. For GPU results to be used interchangeably with existing CPU algorithms, the GPU algorithm should produce results that are reproducible and convergent with results obtained by the CPU algorithm. For example, Hernandez-Fernandez et al., compared the mean of a few representative diffusion weighted voxel values in a repeated test between CPU and GPU and found almost identical results [6]. However, their study did not report on CPU/GPU differences in contiguous within-slice voxels or multi-slice brain data. The current study aims to extend these findings by comparing sampled distribution shapes of CPU and GPU Bayesian estimation of diffusion parameters in a whole brain dataset.

This paper is organized as follows. Brief introductions of diffusion MRI and Bayesian estimation of diffusion parameters are given. Then, the complete methodology of output comparison technique is described. Results of output comparison are presented for each diffusion parameter type, and then, we give our conclusions and discussions

### Diffusion MRI and bayesian estimation

Diffusion MRI (dMRI) is a useful tool in visualizing the white matter connectivity of the brain and is widely used in both research and clinical contexts. dMRI is sensitive to molecular diffusion of water and enhances the anisotropy—the directional dependence—of neuronal white matter fibre tracts, which can be used to create fractional anisotropy maps, mean diffusivity maps and fibre pathways [18, 19]. A commonly used method to estimate the fibre orientations and reconstruct the brain tracts in vivo is to use the FMRIB Software Library’s (FSL) “Bayesian estimation of diffusion parameters” (*bedpostx*) and “probabilistic tracking of crossing fibres” (*probtrackx*) algorithms. In brief, *bedpostx* employs a Markov Chain Monte Carlo sampling technique to estimate the posterior probability density functions (PDF) of the diffusion parameters utilizing the “ball-and-stick” model which takes into account multiple fibre orientations in a given voxel where appropriate [11, 12]. This allows the resolving of within-voxel fibre crossings, which is a common hurdle during the fibre tracking step, by fitting more than one fibre orientation in a given voxel only when it is relevant to do so. This feature is the “automatic relevance determination” (ARD) algorithm in *bedpostx* [11] which initially sets the additional fibre fractions in secondary orientations to zero with low variance, and iteratively estimates the variance separately so that when the additional fibre orientation is supported by the data, the additional fibre fraction can take a non-zero value with a larger variance. *bedpostx* uses the Levenberg-Marquardt (L-M) fit to initialize parameters by minimizing the sum of squared model residuals, similar to fitting a diffusion tensor model, then, it proposes a value for each parameter, drawing from Normal proposal distribution, calculates the likelihood term, and accepts or rejects the proposed value based on a Metropolis acceptance criterion. When employing the ball-and-stick model where the isotropic compartment is fitted with a mean value within a voxel (i.e. model = 1), *bedpostx* gives the following PDF distributions for each voxel as output: diffusivity value (*d)*, baseline signal (*S0*), weight of each fibre orientation’s contribution to anisotropic diffusion signal (stick), also known as fibre fraction values (*f*_1_, *f*_2_, etc.), and each fibre orientation’s directional angles expressed in polar coordinates (ϕ_1_, θ_1_, ϕ_2_, θ_2_, etc). These PDFs are then randomly sampled by *probtrackx* to create fibre streamlines through stochastic propagation of multiple particles through the diffusion space [11, 12]. Because *bedpostx* processes each voxel serially in the CPU, an extensive amount of computational time is required to obtain the PDFs, which makes it impractical for utilization in a clinical medical environment where a computing cluster may not be available [20, 21]. To alleviate this problem, and to reduce computational time substantially FSL provides a GPU-based parallelized version of *bedpostx*, called *bedpostx_gpu* [6]. Here, the L-M initialization and MCMC sampling are parallelized such that multiple voxels are processed simultaneously. Difference in operation order exists between *bedpostx* and *bedpostx_gpu* such that, in the GPU, L-M initialization for the entire brain is done first, then MCMC sampling are done for the entire brain, whereas in the CPU, L-M initialization and MCMC sampling are done in sequential order for each voxel. We know of no study to date that has quantitatively examined output similarities and differences between the *bedpostx* and *bedpostx_gpu* algorithms in a whole-brain DTI dataset. Further, because the PDF distributions obtained from *bedpostx* is used in obtaining fibre streamlines in *probtrackx* and not their mean values, differing distributional shapes between the two algorithms can also cause bias in output fibre tracking using *probtrackx*. This study aims to compare the output of *bedpostx* and *bedpostx_gpu* and report on output PDF distribution (*f*_1_, *f*_2_, ϕ_1_, θ_1_, ϕ_2_, θ_2_) shape difference, magnitude of difference in mean value and underlying uncertainty value.

## Methods

### Computational resources

*bedpostx* was used for output comparison with the GPU version *bedpostx_gpu*, both from FSL 6.0.5 package running on Ubuntu 20.04 LTS. The CPU version ran on a workstation with a dual Intel Xeon X5670 2.93 GHz CPU with 6 x 4-GB DDR3-1333 memory, and 24 threads. The GPU version ran on a workstation with one NVIDIA Tesla C2075 with 448 CUDA cores, 6-GB GDDR5 dedicated memory, PCIe x16 bus, CUDA 8.0 with driver version 390.144.

### Data

Diffusion data from the MGH-USC Human Connectome Project (HCP) Image & Data Archive portal (https://ida.loni.usc.edu/), subject: mgh1005, was used for running multiple trials of *bedpostx* and *bedpostx_gpu*. The full dMRI data consist of directional volumes acquired in multiple shells (b = 0,1000,3000,5000,10000) but for our work, a single shell from the full set was used for analysis: motion and eddy corrected, b = 1000, 64 directional volumes and 6 non-directional volumes, 1.5mm isotropic, 140x140x96. This was chosen because most clinical and research studies have access to a similar single-shell dMRI acquisition method and the resulting data can still support multi-fibre modeling of *bedpostx* algorithms. T1-weighted anatomical scan of the same subject was segmented [22] to derive binary masks of grey matter, white matter and cerebrospinal fluid, then co-registered to the diffusion data [23, 24]. These masks were used to quantify how many significantly different distributions were localized in each tissue class. Analysis was repeated on a different HCP subject data, mgh1001, to assess reproducibility of comparison results.

In the absence of ground truth data, a whole brain synthetic dMRI data was generated using the ball-and-stick model through Dipy 0.16.0 [25] by the following. First, a single run of *bedpostx* on the CPU was done on subject mgh1005’s dMRI data to produce mean values of diffusion parameters *d*, *S0*, *f*_1_, *f*_2_, ϕ_1_, θ_1_, ϕ_2_, θ_2_. Then, these mean values were used to recreate a simulated dMRI data with two fibre fractions and mean principal diffusion directions for each voxel. Finally, the simulated dMRI data was used to run bedpostx on CPU and GPU 20 times to generate 1000 samples of diffusion parameters with the goal of comparing the resulting sample mean values against the input mean values used for creating the simulated data. The random number generator seed used for generating the simulated data was different than to those used to generate these samples.

### *bedpostx* PDF creation

Specified *bedpostx* and *bedpostx_gpu* input parameters are: 2250 MCMC iterations, of which during the latter 1250 iterations, parameter values were recorded to PDF every 25 iterations, resulting in 50 samples per PDF; monoexponential model (i.e. fit with mean diffusivity) with ARD fitting 2 fibres per voxel where appropriate. 20 trials of *bedpostx* and *bedpostx_gpu* were run with different random number generator seeds and output distributions from all trials were merged together to form 1000 samples per parameter PDF for *bedpostx* and for *bedpostx_gpu*. Furthermore, to inspect differences in L-M initialization between *bedpostx* and *bedpostx_gpu*, 20 trials of each algorithm were run again but with 1 iteration to record 1 sample close to the initializing value.

### PDF distribution comparison and statistical analysis

PDF shape was statistically compared via two-sample Kolmogorov-Smirnov (KS) test to derive voxels that have different distributions between CPU and GPU (two-tailed, p < 0.05, uncorrected). Family-wise error rate was controlled by the Bonferroni method [26]. Voxels with significantly different distributions were then further categorized by their KS-scores (S) in 4 different ranges: 0.1–0.2, 0.2–0.3, 0.3–0.4, > 0.4. S scores illustrate the amount of sample deviation (e.g. S = 0.35, 35% of samples differ between two distributions). For *f*_1_ and *f*_2_, CPU mean values along with absolute difference in mean CPU/GPU values were calculated and averaged for each S range. For angles, mean, standard deviation and median difference in principal diffusion directions (PDD) along with 95^th^-percentile cones of angular uncertainties (CAUs) were calculated in voxels with at least one significantly different angle parameter for each pairs (i.e. ϕ_1_ OR θ_1_, ϕ_2_ OR θ_2_). Maximum S score between the [ϕ,θ] pair was used when categorizing significantly different angle parameters into different S-ranges.

### Effect of mixed fibre fraction and orientation samples near crossing-fibre areas

Fibre fraction parameters (*f*_*1*_, *f*_*2*_, etc.) and their associated orientations (ϕ_1_θ_1_,ϕ_2_θ_2,_ etc.) could be inconsistently associated with the different underlying sub-fibre populations, especially if the fibre fractions are of comparable strength [27]. This can cause differing proportions of fibre fraction and orientation values to be labeled as one group (e.g. *f*_*1*_,ϕ_1_θ_1_) but labeled as another on the next trial (e.g. *f*_*2*_,ϕ_2_θ_1_). There is no guarantee that the labeling happens consistently and because we are merging samples from 20 different *bedpostx* and *bedpostx_gpu* trials to form the PDF distributions for comparison, it is possible that differences between the two platforms occur due to the this inconsistent labeling of sub-fibre populations. To investigate this effect of mixed fibre fractions and how much it may contribute to CPU and GPU output differences, we swapped *f*_*1*_,ϕ_1_θ_1_ and *f*_*2*_,ϕ_2_θ_2_ where *f*_*2*_ > *f*_*1*_ and ran the same statistical analysis on the swapped samples and compared the results against statistically different unswapped samples.

## Results

### Difference in L-M initialization

**Fig 1:**
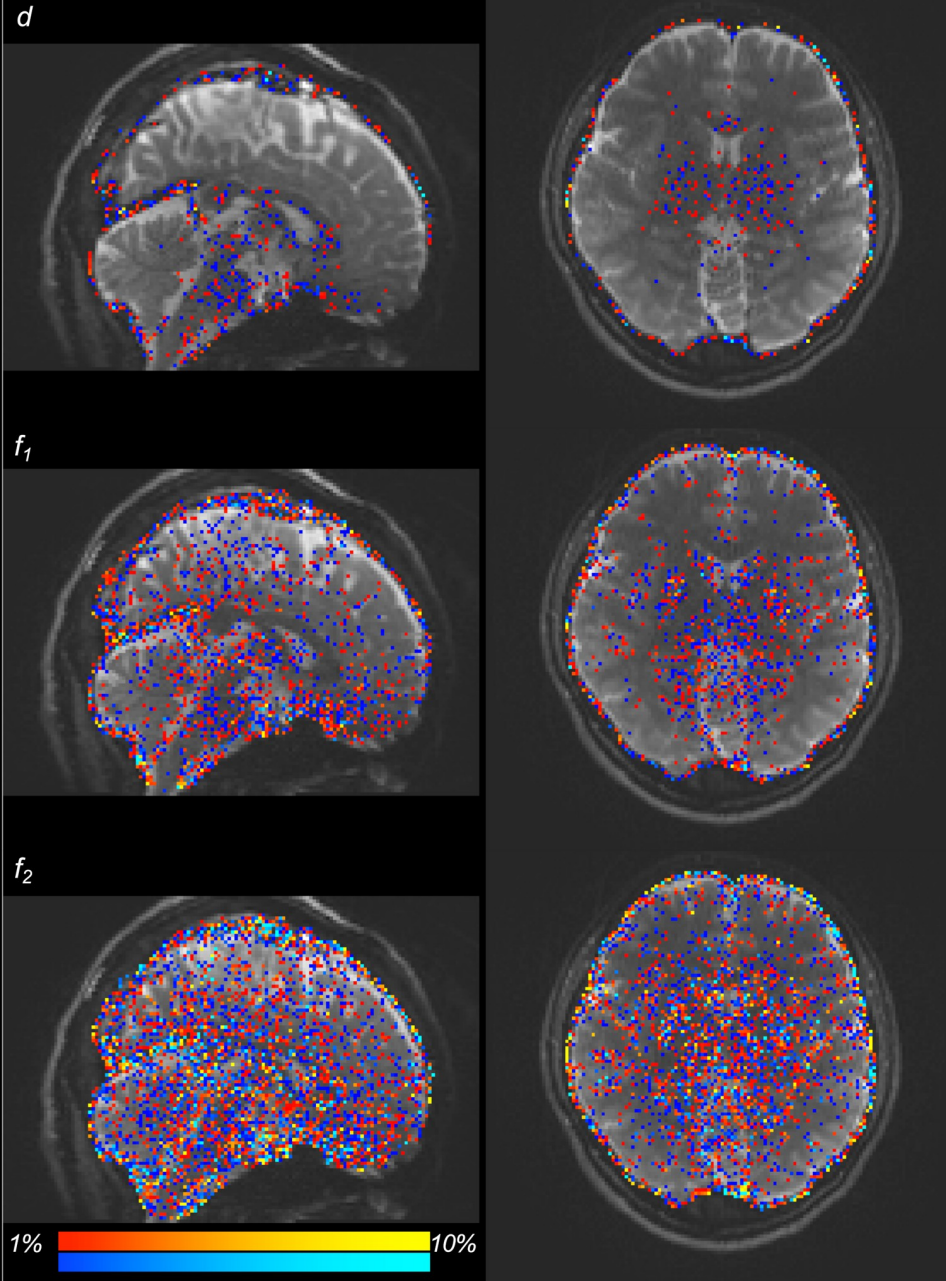
CPU and GPU L-M initialization difference map. L-M initialization difference between CPU and GPU. Orange-yellow colors are CPU > GPU regions, and blue-light blue colors are GPU > CPU regions. Difference in scalar maps are thresholded at a magnitude of 1% with respect to mean CPU values.

Example L-M initialization difference map is shown in Fig 1 with difference greater than 1% of mean CPU values color coded. Diffusivity and baseline signal (*d*, *S0)* have increased difference towards the center of the brain, and *f*_*2*_, ϕ_2_, and θ_2_ have greater amount of different voxels compared to *f*_*1*_, ϕ_1_ and θ_1_.

### f_1_

**Fig 2:**
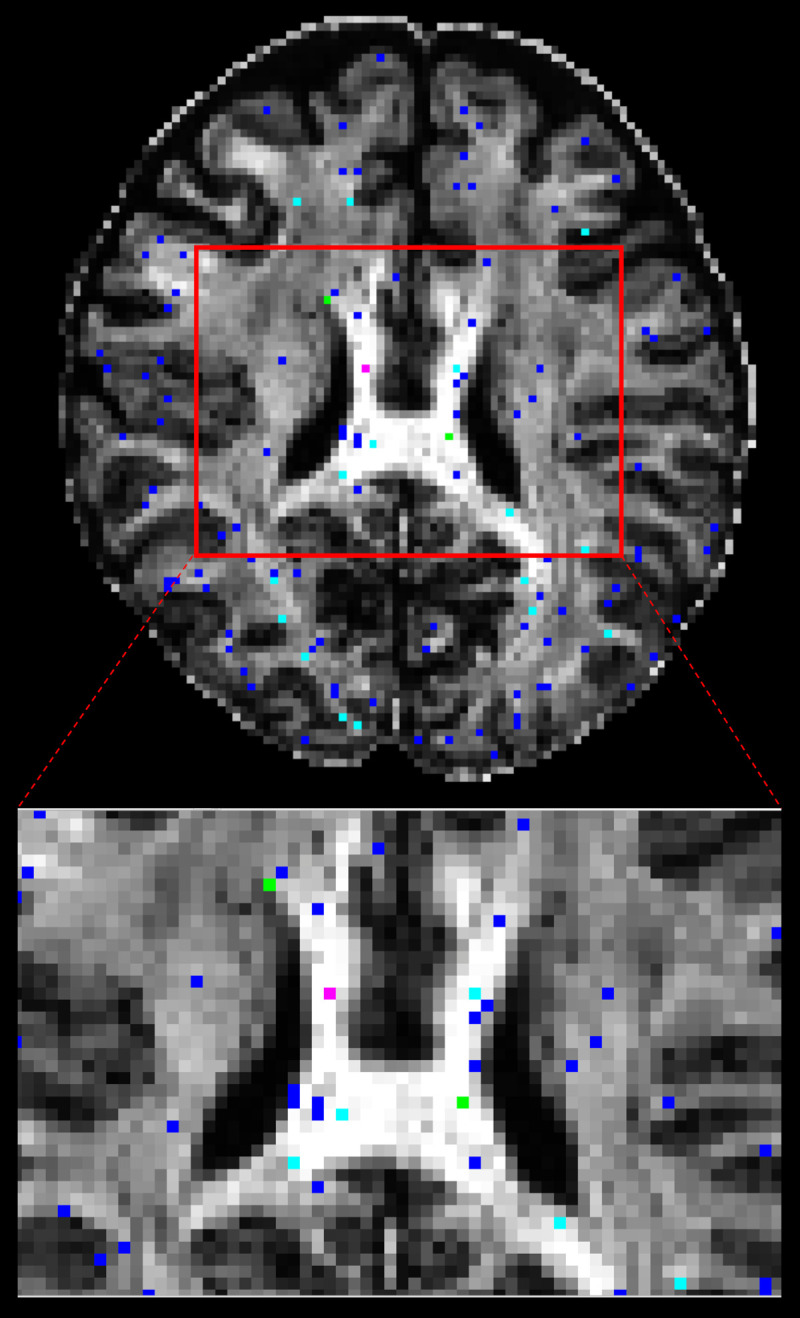
Significantly different *f*_1_ map. Significantly different f_1_ overlaid on mean f_1_ image. S-score ranges are in: 0.1–0.2 = blue, 0.2–0.3 = light blue, 0.3–0.4 = green, > 0.4 = magenta (enlarged view from area in red-square).

About 1% of total number of brain voxels (5145 of 436738) had significantly different *f*_*1*_ distributions. Significantly different voxels were sparsely localized throughout the brain bilaterally. Of the significantly difference voxels, 1% were found outside the three tissue class binary masks, 2% were found in cerebrospinal fluid, 34% were found in grey matter and 63% were found in white matter. The latter were located in long white matter projections, such as corpus callosum, corona radiata, internal capsules and anterior and posterior thalamic radiations (Fig 2). Number of significant voxels, mean CPU *f*_*1*_, absolute average differences in mean *f*_*1*_, and absolute average difference in L-M initialization in each S-score regions are summarized in Table 1.

**Table 1:** Significantly different *f*_*1*_ distributions.

*S score (# of voxels)*	*Average Mean f*_*1*_ *CPU (stdev)*	*Average |Mean f1 CPU–Mean f1 GPU|*	*Average |L-M CPU–L-M GPU|*
*0.1–0.2 (4487)*	*0.3320 (0.1606)*	*0.0179*	*0.0008*
*0.2–0.3 (614)*	*0.4061 (0.1555)*	*0.0337*	*0.0007*
*0.3–0.4 (42)*	*0.4457 (0.1656)*	*0.0572*	*0.0196*
*> 0.4 (2)*	*0.6459 (0.4800)*	*0.1439*	*0.1972*
*All*	*0.3419 (0.1623)*	*0.0202*	*0.0010*

Significantly different *f*_*1*_ distributions: for each S-score range, averaged mean f_1_ of CPU distributions, averaged absolute difference in mean *f*_*1*_ and averaged absolute difference in L-M initialization are tabulated.

**Fig 3:**
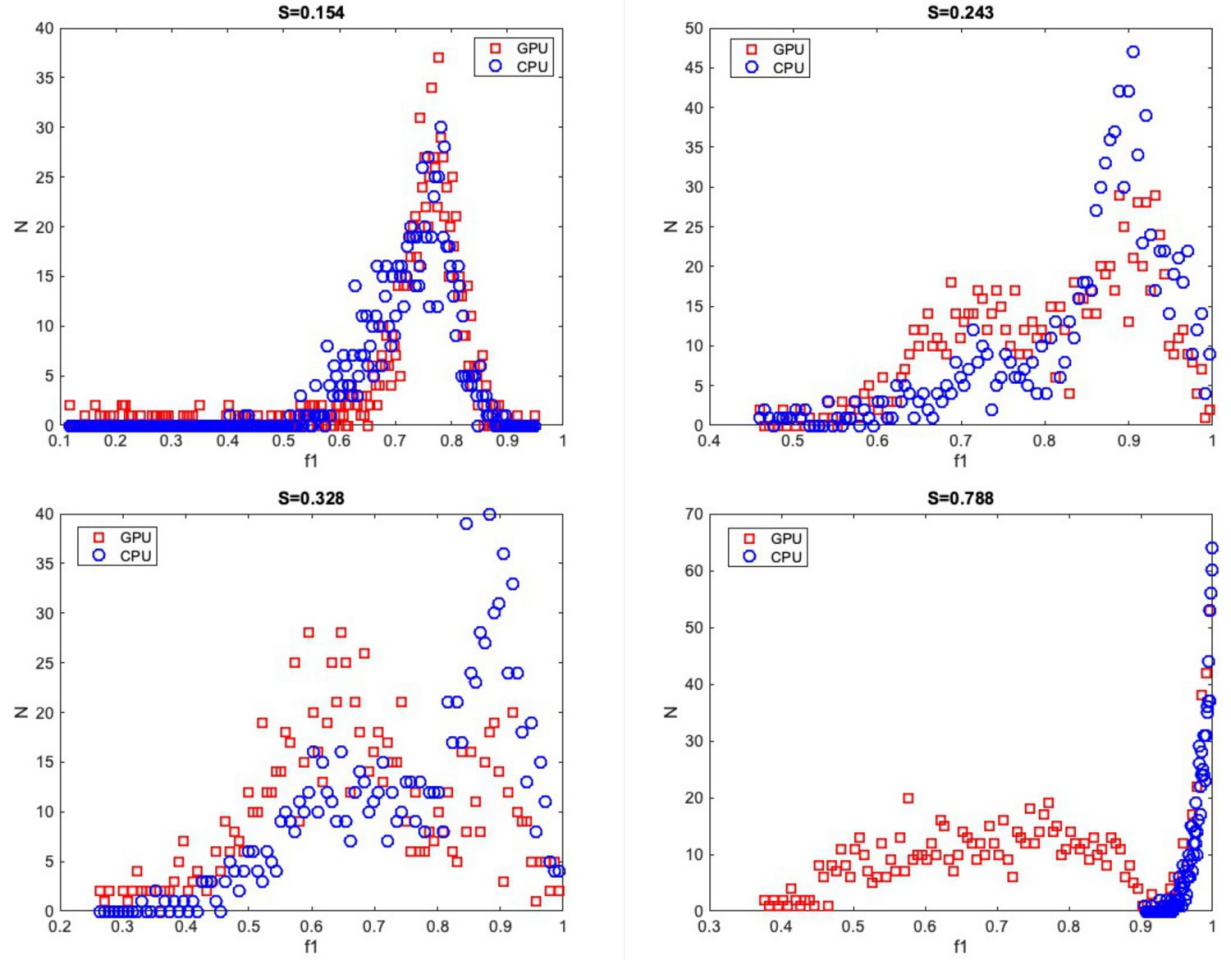
Example PDF distribution shape difference of *f*_*1*_. Example PDF distribution shape differences of significantly different *f*_*1*_.

Majority of voxels had S-scores less than 0.3 (5101 out of 5139). Example PDF distribution shapes of CPU and GPU in significant voxels are shown in Fig 3. The largest S-score of 0.788 was found in the body of corpus callosum. Here, both *f*_1_ distributions have peaks near 0.99. CPU data had a sharper peak, with all samples above 0.9. Fewer GPU samples are above 0.9, with the remainder between 0.4 and 0.9. Here, average *f*_*1*_ initialization across 20 trials by CPU L-M was 0.998 while average *f*_*1*_ initialization by the GPU L-M across 20 trials was 0.605. Larger average L-M initialization differences were noted for larger S-score. After adjusting for *f*_*2*_>*f*_*1*_ samples by swapping, 740 voxels were no longer significantly different.

Similar results were found on another subject with significantly different *f*_*1*_ voxels sparsely located throughout the whole brain. 4128 voxels were different with an average difference in magnitude at 0.0257. The magnitude of differences in mean *f*_*1*_ values in CPU and GPU were 24.23% and 24.25% of simulated *f*_*1*_ values respectively in the whole brain. Within the whitematter only, the differences were both 8.27% of simulated *f*_*1*_ values.

### f2

31% of total number of brain voxels (137240 out 436738) had significantly different *f*_*2*_ distributions. Significantly different distributions were localized in grey matter (50%), cerebrospinal fluid (19%) and white matter (18%). For the white matter, they were localized in long white matter projections similar to those identified in *f*_1_.

89% of significant voxels had CPU or GPU mean *f*_2_ values lower than 0.05, predominantly in areas with grey matter and cerebrospinal fluid, likely the effect of ARD estimating *f*_*2*_ to zero in both *bedpostx* and *bedpostx_gpu*. To focus analysis on areas where *f*_*2*_ is supported by data, we reported mean CPU *f*_*2*_, absolute average differences in mean *f*_*2*_, and absolute average difference in L-M initialization in each S-score regions only on areas with mean *f*_2_ from CPU or GPU greater than or equal to 0.05 (Table 2).

**Table 2:** Significantly different *f*_*2*_ distributions.

*S score (# of voxels)*	*Average Mean f*_*2*_ *CPU (stdev)*	*Average |Mean f2 CPU—Mean f2 GPU|*	*Average |L-M CPU—L-M GPU|*
*0.1–0.2 (10390)*	*0.1211 (0.0576)*	*0.0206*	*0.0010*
*0.2–0.3 (3602)*	*0.1082 (0.0477)*	*0.0344*	*0.0011*
*0.3–0.4 (406)*	*0.1073 (0.0490)*	*0.0550*	*0.0012*
*> 0.4 (25)*	*0.1051 (0.0428)*	*0.0753*	*0.0104*
*All*	*0.1175 (0.0554)*	*0.0252*	*0.0010*

Significantly different *f*_*2*_ distributions where mean f_2_ in CPU or GPU > 0.05: for each S-score range, averaged mean *f*_*2*_ of CPU distributions, averaged absolute difference in mean *f*_*2*_ and averaged absolute difference in L-M initialization are tabulated

**Fig 4:**
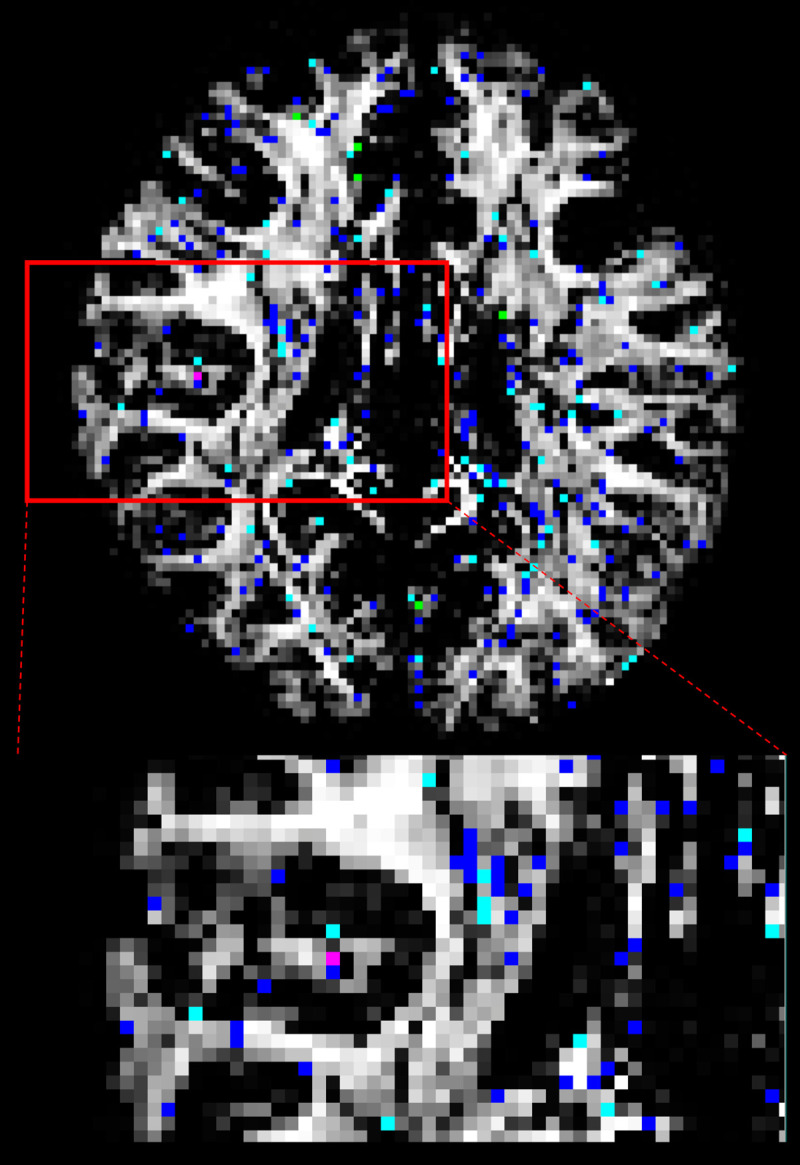
Significantly different *f*_*2*_ map where *f*_*2*_>0.05. Significantly different *f*_*2*_ with mean *f*_*2*_ in CPU or GPU > 0.05. S-score ranges are in: 0.1–0.2 = blue, 0.2–0.3 = light-blue, 0.3–0.4 = green, > 0.4 = magenta (enlarged view from area in red-square).

This was the same threshold chosen by [11] when looking for secondary fibre orientations supported by ARD (Fig 4). Here, most significantly different voxels were localized in grey/white matter junctions. Some were sparsely found bilaterally within identifiable structures such as corpus callosum, corona radiata, internal capsule, anterior and posterior thalamic radiations.

**Fig 5:**
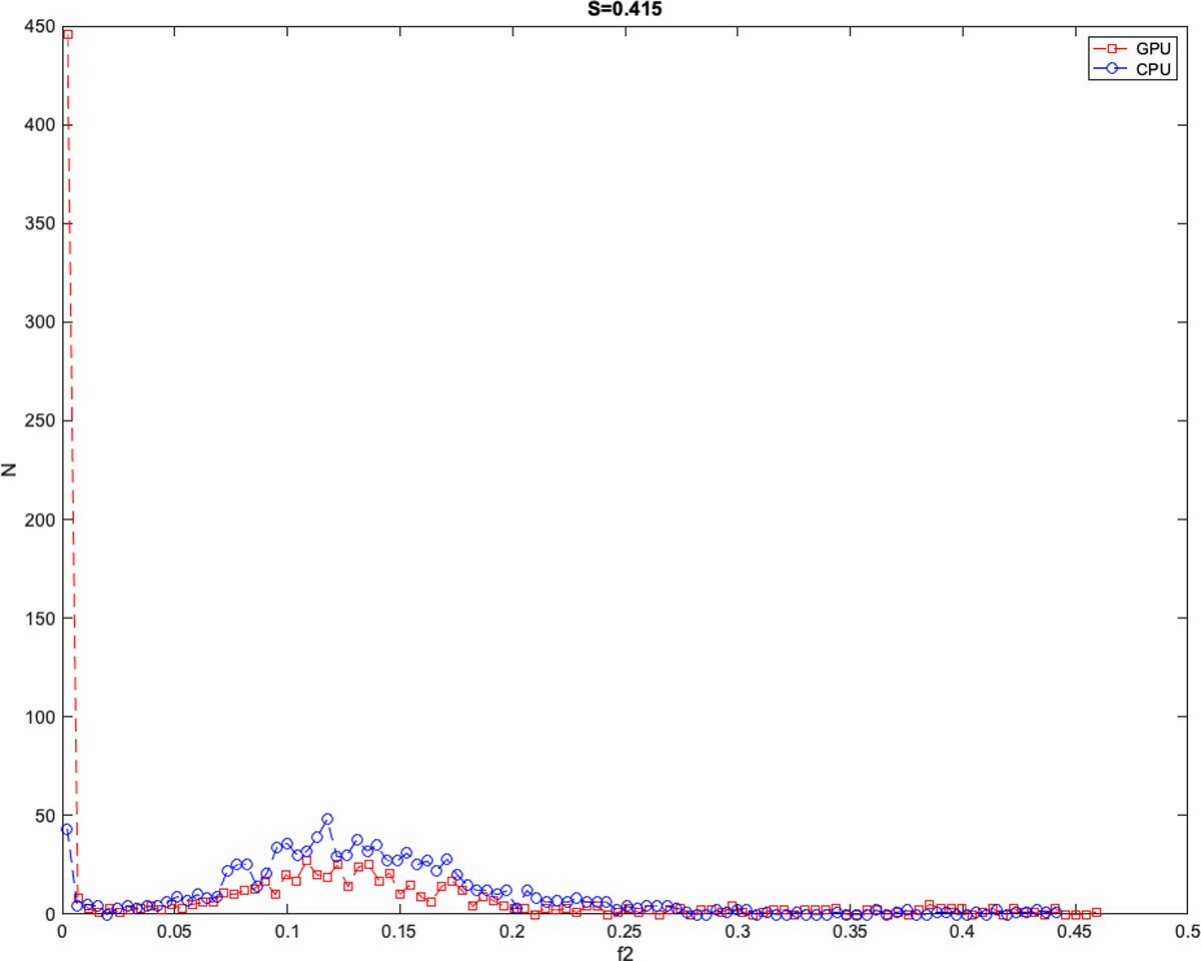
Example distribution shape difference in *f*_*2*_. Example distribution shape differences in *f*_*2*_. Red squares with dotted line denotes GPU samples and blue circles with dotted line denotes CPU samples.

The majority of significant *f*_2_ distribution differences had S-scores < 0.3 (13992 out of 14423). One example of a voxel exhibiting a large PDF difference in *f*_2_ is depicted in Fig 5, where S-score = 0.415 which is the same location as the largest S-score found in Fig 4 (magenta).

Here, the distribution shows a higher peak near *f*_2_ = 0 in the GPU distribution and lower counts of samples between 0.05 and 0.2. In the CPU, there is a smaller peak near *f*_*2*_ = 0 with a larger counts of samples between 0.05 to 0.2. The CPU L-M initialization step estimated *f*_*2*_ = 0.143 averaged across 20 trials while GPU L-M initialization step had *f*_*2*_ = 0.273 across 20 trials. Similar to *f*_*1*_, larger average L-M differences were found for larger S ranges. After adjusting for *f*_*2*_>*f*_*1*_ samples, 836 voxels were no longer significantly different, and of these voxels, 680 were in areas with mean *f*_2_ from CPU or GPU greater than or equal to 0.05.

Similar results were found in another subject where total number of significantly different *f*_*2*_ voxels with mean *f*_*2*_ > 0.05 was 15129, and mean magnitude difference at 0.0302. The magnitude of differences in mean *f2* values in CPU and GPU were both 35.79% of simulated *f*_*2*_ values where simulated *f*_*2*_ > 0.05. Within the whitematter only, the differences were 17.98% and 18.00% of simulated *f*_*2*_ for CPU and GPU, respectively.

### ϕ_1_ and θ_1_

**Fig 6:**
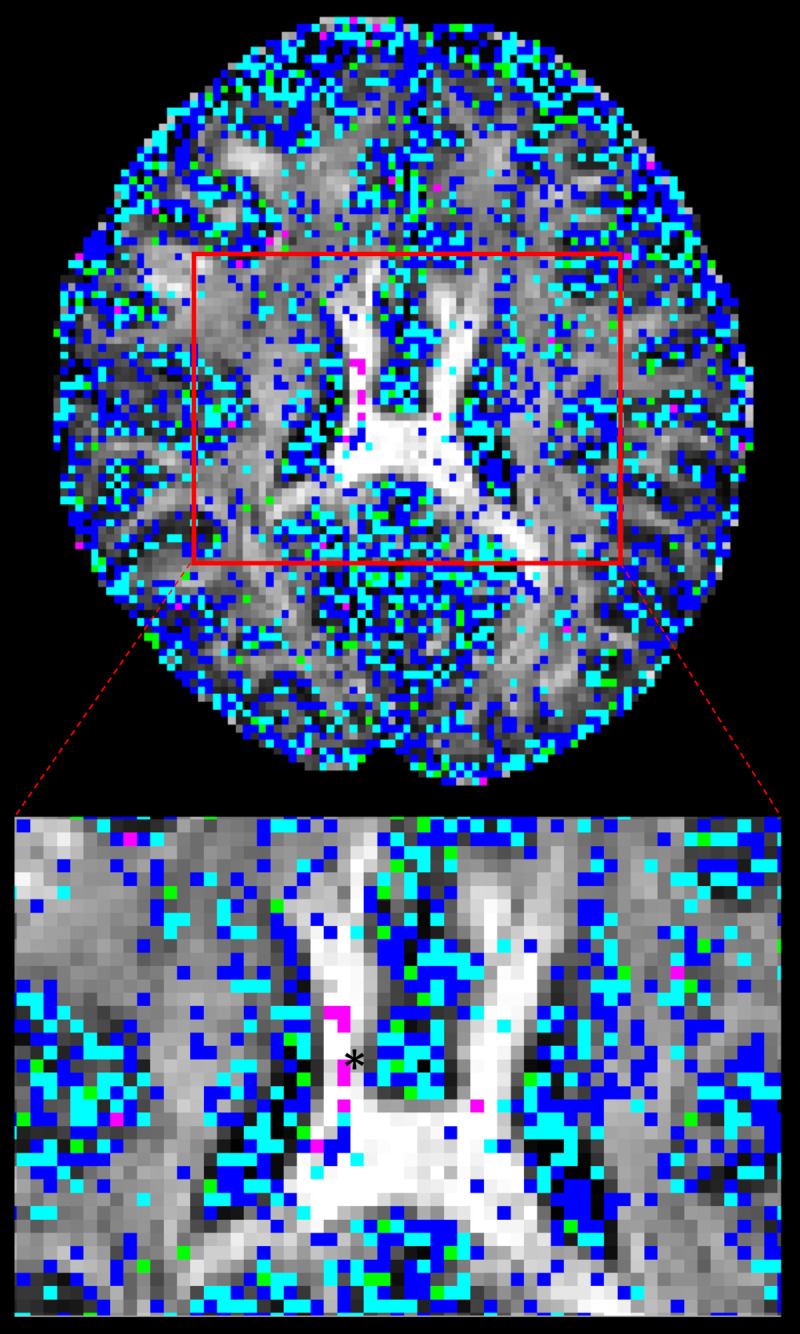
Significantly different ϕ_1_ and θ_1_. Significantly different ϕ_1_ and θ_1_ distributions with S-ranges in 0.1–0.2 (Blue), 0.2–0.3 (light-blue), 0.3–0.4 (green), > 0.4 (magenta). Maximum S-score between ϕ_1_ and θ_1_ was used to categorize each location into different range (enlarged view from area in red-square).

198166 out of 436738 total brain voxels had significantly different ϕ_1_ or θ_1_ distribution. Significantly different distributions were localized predominantly in areas of grey matter (50%) and cerebrospinal fluid (21%). They were also found in the white matter (15%), with some key white matter structures such as corpus callosum, internal capsules, corona radiata and anterior and posterior thalamic radiations containing significantly different distributions (Fig 6).

Mean and median angle differences, and average 95^th^ percentile CAUs for each S-score range are tabulated in Table 3.

**Table 3:** Significantly different ϕ_1_, θ_1_.

*S score (# of voxels)*	*Average Δmean PDD*	*Stdev Δmean PDD*	*Median Δmean PDD*	*Mean 95* ^*th*^ *-percentile CAU*	
				*CPU*	*GPU*
*0.1–0.2 (112319)*	*2.186°*	*4.261°*	*1.053°*	*51.126°*	*51.092°*
*0.2–0.3 (71395)*	*2.186°*	*4.107°*	*1.075°*	*55.523°*	*55.523°*
*0.3–0.4 (12840)*	*2.280°*	*4.465°*	*1.080°*	*58.256°*	*58.307°*
*> 0.4 (1612)*	*2.482°*	*5.508°*	*1.055°*	*57.509°*	*57.855°*
*All*	*2.194°*	*4.232°*	*1.064°*	*53.224°*	*53.211°*

For each S-score range, average of mean PDD difference, standard deviation of mean PDD difference, median of mean PDD difference and 95^th^-percentile cone of angular uncertainty are tabulated.

Again, the majority of these voxels have S < 0.3 (183714 out of 196081). Mean difference in angles of principle diffusion directions in all significantly different voxels was 2.194° (stdev = 4.232°) while the median difference was 1.064°. In all significantly different ϕ_1_ and θ_1_ voxels, the average angular difference between the 95^th^ percentile CAUs for CPU and GPU is 0.013° (CPU 53.224°; GPU 53.211°; see Table 3). Because ϕ_1_ and θ_1_ parameters are more meaningful in white-matter where anisotropy is higher, angular differences and 95^th^ percentile CAUs for each S-score range in white-matter only are tabulated in Table 4.

**Table 4:** Significantly different ϕ_1_, θ_1_ in the white-matter.

*S score (# of voxels)*	*Average Δmean PDD*	*Stdev Δmean PDD*	*Median Δmean PDD*	*Average 95* ^*th*^ *-percentile CAU*	
				*CPU*	*GPU*
*0.1–0.2 (18753)*	*2.286°*	*3.297°*	*1.285°*	*37.973°*	*37.991°*
*0.2–0.3 (8996)*	*3.316°*	*4.895°*	*1.586°*	*44.925°*	*44.889°*
*0.3–0.4 (1419)*	*4.890°*	*7.303°*	*1.715°*	*48.315°*	*48.240°*
*> 0.4 (188)*	*6.052°*	*11.896°*	*1.066°*	*40.770°*	*41.438°*
*All*	*2.751°*	*4.277°*	*1.381°*	*40.621°*	*40.622°*

For each S-score range, average of mean PDD difference, standard deviation of mean PDD difference, median of mean PDD difference and 95^th^-percentile cone of angular uncertainty are tabulated.

**Fig 7:**
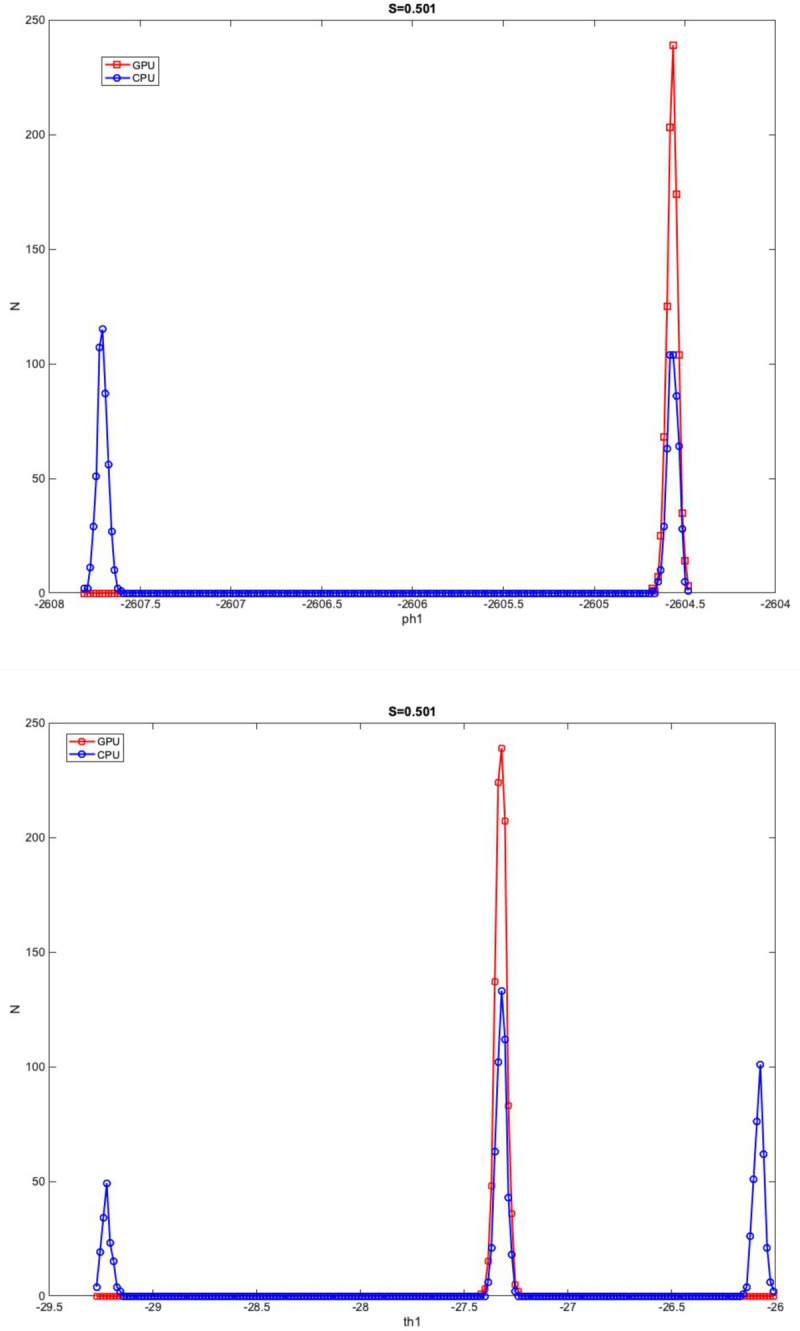
Distribution shape of ϕ_1_ and θ_1_. Distributions of ϕ_1_ and θ_1_ that are significantly different were derived from one representative voxel with a particularly high S-score of 0.501. The voxel was located within the corpus callosum. Red squares and line denote GPU samples while blue circles and line denote CPU samples.

**Fig 8:**
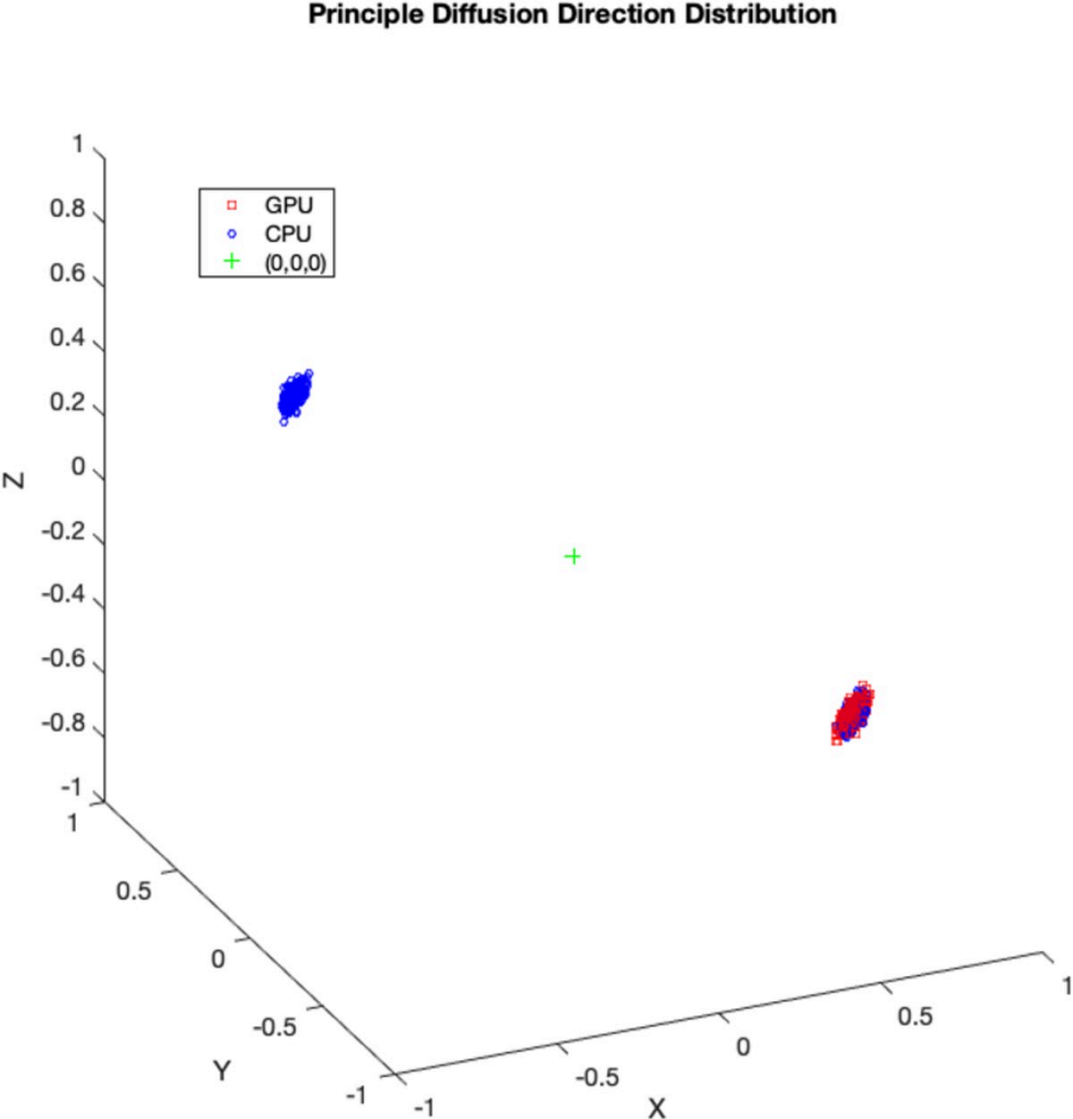
Distribution of principal diffusion directions. The effect of distribution shape difference in ϕ_1_, θ_1_ samples on principal diffusion directions is illustrated in 3d plot. Red squares denote GPU samples while blue circles denote CPU samples.

Overall, higher average difference in mean PDD and lower CAUs were found in significantly different voxels confined to the white-matter. An example distributions of ϕ_1_ and θ_1_ is shown in Fig 7. The depicted distributions in Fig 7 came from a voxel located in the corpus callosum (marked by * in Fig 6.) with S score = 0.501. In this voxel, two sharp peaks were found for CPU ϕ_1_ samples whereas only a single peak was found for GPU whose location coincided with one of CPU’s peak values. For θ_1_ samples, GPU only had one sharp peak, whereas CPU had three peaks, one of which was similar to the GPU. The effect of distribution shape difference on diffusion direction is illustrated in Fig 8. It is noted that while the GPU principal diffusion directions are all estimated similarly, CPU principal diffusion directions have two separate clusters that are located antiparallel to each other.

Adjusting for *f*_*2*_ *> f*_*1*_, 18231 voxels became not significantly different and of these, 6755 were in the white-matter.

Similar results were found in another subject’s data with 190602 significantly different ϕ_1_,θ_1_ voxels with average mean PDD difference 2.559°, with 95-percentile CAUs of 38.943° and 38.838° for CPU and GPU respectively. Compared to simulated data’s mean principal direction, both CPU and GPU produced 8.2° mean difference and 3.9° median difference in the white matter.

### ϕ_2_ and θ_2_

**Fig 9:**
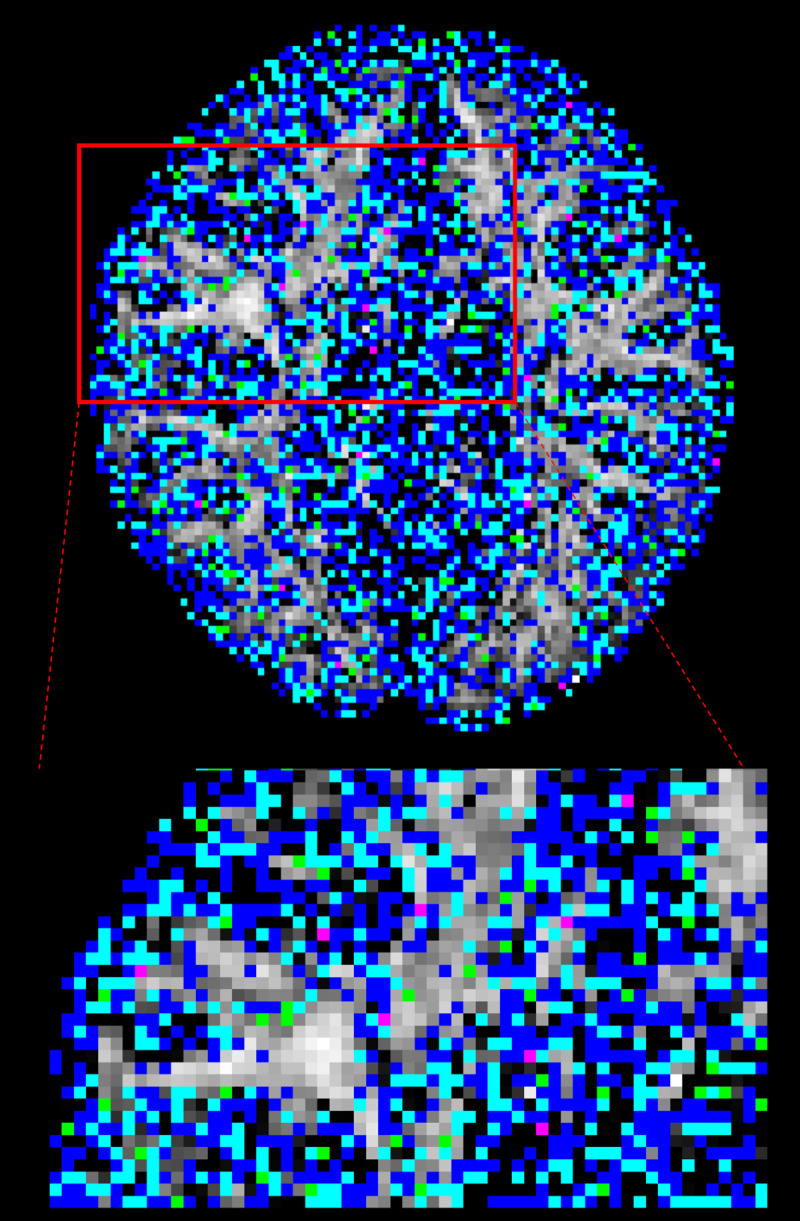
Significantly different ϕ_2_ and θ_2_. Significantly different ϕ_2_ and θ_2_ distributions with S-ranges in 0.1–0.2 (Blue), 0.2–0.3 (light-blue), 0.3–0.4 (green), > 0.4 (magenta). Maximum S-score between ϕ_2_ and θ_2_ was used to categorize each location into different range (enlarged view from area in red-square).

224337 out of 436738 total brain voxels had significantly different ϕ_2_ or θ_2_ distributions. Significantly different distributions were localized in grey matter (51%), cerebrospinal fluid (16%) and white matter structures (22%) such as corpus callosum, corona radiata, internal capsule, and the anterior and posterior thalamic radiations (Fig 9). Mean and median angle differences along with 95^th^ percentile CAUs for each S-score range are tabulated in Table 5. Again, most voxels have S-scores < 0.3 (213437 out of 223309).

**Table 5:** Significantly different ϕ_2_, θ_2_.

*S score (# of voxels)*	*Average Δmean PDD*	*Stdev Δmean PDD*	*Median Δmean PDD*	*Average 95* ^*th*^ *-percentile CAU*	
				*CPU*	*GPU*
*0.1–0.2 (139329)*	*37.222°*	*29.403°*	*34.915°*	*83.215°*	*83.217°*
*0.2–0.3 (74108)*	*35.832°*	*29.359°*	*31.452°*	*83.954°*	*83.944°*
*0.3–0.4 (10184)*	*37.399°*	*29.434°*	*34.635°*	*83.899°*	*83.963°*
*> 0.4 (716)*	*38.699°*	*29.378°*	*36.395°*	*82.968°*	*82.964°*
*All*	*36.776°*	*29.397°*	*33.798°*	*83.490°*	*83.490°*

For each S-score range, average of mean PDD difference, standard deviation of mean PDD difference, median of mean PDD difference and 95^th^-percentile cone of angular uncertainty are tabulated.

Overall mean difference in principle directions is 36.776° with median difference of 29.397°. Average angular uncertainty is 83.490° for both CPU and GPU.

With ϕ_2_ and θ_2_, it is more meaningful to focus on white matter and areas where *f*_*2*_ > 0.05 (i.e. where ARD has deemed appropriate to fit a second fibre orientation). Mean PDD difference and 95^th^-percentile CAUs in the white-matter and f_2_ > 0.05 for each S-score range are tabulated in Table 6.

**Table 6:** Significantly different ϕ_2_, θ_2_ in the white-matter.

*S score (# of voxels)*	*Average Δmean PDD*	*Stdev Δmean PDD*	*Median Δmean PDD*	*Average 95* ^*th*^ *-percentile CAU*	
				*CPU*	*GPU*
*0.1–0.2 (16560)*	*5.718°*	*9.812°*	*2.617°*	*69.024°*	*69.076°*
*0.2–0.3 (8736)*	*7.715°*	*11.949°*	*3.481°*	*73.807°*	*73.771°*
*0.3–0.4 (1210)*	*11.600°*	*16.677°*	*4.492°*	*73.100°*	*73.226°*
*> 0.4 (87)*	*14.084°*	*20.294°*	*4.151°*	*71.539°*	*70.290°*
*All*	*6.668°*	*11.092°*	*2.924°*	*70.789°*	*70.811°*

**Fig 10:**
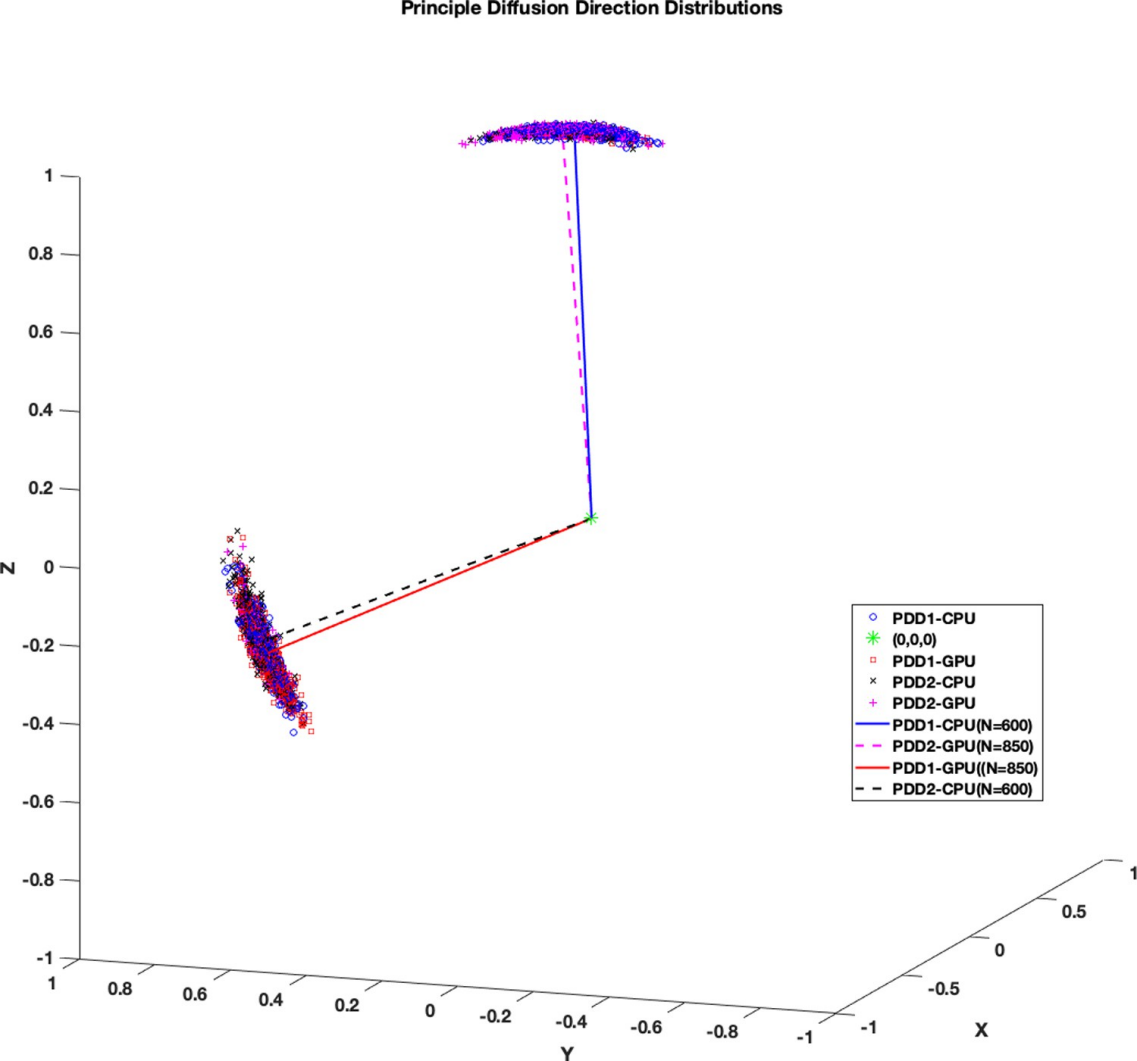
Distribution shape and diffusion directions of ϕ_1_ and θ_1_. Example ϕ_1_,θ_1_ and ϕ_2_,θ_2_ principal diffusion direction distributions from a representative voxel plotted in 3D (Blue circle: CPU ϕ_1_,θ_1_ samples, Red square: GPU ϕ_1_,θ_1_ samples, Black x’s: CPU ϕ_2_,θ_2_ samples, Magenta +’s: GPU ϕ_2_,θ_2_ samples). Mean directions derived from each clusters with majority of samples are represented as line drawings (Blue solid line: CPU ϕ_1_,θ_1_ mean direction, N = 600, Red solid line: GPU ϕ_1_,θ_1_ mean direction, N = 850, Black dotted line: CPU ϕ_2_,θ_2_ mean direction, N = 600, Magenta dotted line: GPU ϕ_2_,θ_2_ mean direction, N = 850).

Like ϕ_1_ and θ_1_, the CAUs were lower when focusing in on the white matter region. Also, the difference in mean PDD was lower for each S-score range. Distributions of PDDs derived from ϕ_2_, θ_2_ samples as well as ϕ_1_, θ_1_ in a representative voxel with S = 0.453 for ϕ_2_, θ_2_ and S = 0.451 for ϕ_1_, θ_1_ near the right dorsolateral frontal are illustrated in Fig 10. There were two clusters of PDD formed by [ϕ_1_, θ_1_] and [ϕ_2_, θ_2_] for both CPU and GPU. Here, it was observed that majority of CPU’s [ϕ_1_, θ_1_] samples (N = 600) and GPU’s [ϕ_2_, θ_2_] samples (N = 850) had similar PDD distributions (angular difference between average PDDs of 2.04°) while CPU’s [ϕ_2_, θ_2_] samples had similar PDD distribution with GPU’s [ϕ_1_, θ_1_] (angular difference between average PDDs of 1.85°). It was noted that this voxel had mean *f*_*1*_ = 0.32 and mean *f*_*2*_ = 0.31 for both CPU and GPU, indicating that two fibre orientations were viable.

For each S-score range, average of mean PDD difference, standard deviation of mean PDD difference, median of mean PDD difference and 95^th^-percentile cone of angular uncertainty are tabulated for significantly different voxels found in the white-matter where mean *f*_*2*_ > 0.05.

In another subject, similar results were found for ϕ_2_,θ_2_ distributions where 213212 voxels were significantly different with mean PDD difference of 37.161°. In the white matter where mean f2 > 0.05, the mean PDD difference was 7.433° with 75.2° 95^th^-percentile CAUs for both CPU and GPU. Compared to simulated data’s mean principal direction, both CPU and GPU produced 18.4° mean difference and 8.5° median difference in the white matter where simulated *f*_*2*_ values were > 0.05

## Discussions and conclusions

A total of 2620428 pairs of distributions were created and compared across the whole brain. 78% of those distributions showed no significant difference between CPU and GPU. Of the significantly different distributions, 13% were localized outside the three tissue class binary masks, 50% were localized in grey matter, 18% in cerebrospinal fluid, and 19% in white matter, localized within the corpus callosum in the midline, and bilaterally within the corona radiata, internal capsule, and the anterior and posterior thalamic radiations. When analysis was repeated on another subject, similar results were observed. Significantly different *f*_*1*_ distributions in a prominent white-matter structure, such as the body of corpus callosum in the midline as displayed in Fig 2 (magenta label), have been noted with more than half of the samples produced by CPU and GPU differing in value. In general, the corpus callosum contains a well-defined fibre bundle in the Left-to-Right orientation and thus we expected a higher *f*_*1*_ with lower uncertainty. Instead, we observed that CPU estimated all samples to be > 0.9, while the GPU had a larger spread of samples between values of 0.4 and 0.9. Since the corpus callosum in human brain would generally be found near the centre of the whole brain diffusion data, the relative operation order at which this voxel gets processed between the CPU and GPU would have differed greatly (earlier in GPU vs. later in CPU), and this may explain why significant difference was observed even in a prominent structure such as the corpus callosum. We also examined the initialization stages of L-M fit and noted that the results differed between CPU and GPU. For the L-M fit algorithm between CPU and GPU, the difference in operation order of when a particular voxel gets initialized is less impactful since L-M fit simply minimizes sum-of-squared residuals [6]. However, the different CPU and GPU-CUDA math libraries can result in different initialization values. Although GPU-CUDA math operations are double-precision capable [14], increased peak performance (e.g. higher speed-up) is found when single-precision operations are used in their place which may differ in precision compared to CPU math operations [28]. Our findings from Tables 1 and 2 show that larger L-M initialization difference at the start of MCMC sampling results in larger S-scores in significantly different distributions. This suggests that differences in PDF samples appeared to be stemming from a combination of the following: 1) differing starting points after the L-M fit, 2) differing operation order and 3) difference in math library. It is also observed that the L-M initialization difference maps (Fig 1) spatially resemble the inverse of a typical SNR map from a multi-channel MR head-coil [29, 30]. Though we did not directly compare the SNR map of this dataset with L-M differences, we note and speculate that the larger difference in L-M initialization values between CPU and GPU that are mostly found near the inner most structure of the brain could be possible due to less SNR typically found in this region of the brain due to the head-coil SNR profile. This could also possibly explain why large difference was even noted in prominent white matter such as body of corpus callosum: the scan orientation is such that this structure is farther away from the head-coil elements. It could be possible to reduce the amount of differences between the two algorithms by acquiring data with higher SNR as much as possible, or to use DWI post-processing steps to estimate and reduce the noise (e.g. MRTrix *dwidenoise*) from the data before starting the parameter estimation step [31]. In the absence of ground-truth data to compare, we have created simulated ball-and-stick whole brain data using [25] to establish a benchmark error margins in output parameters of CPU and GPU. We found that for all bedpostx parameters, CPU and GPU both produced samples with similar magnitude of differences against the simulated data. This suggests that CPU and GPU would perform similar to each other when estimating against a ground-truth data, if available. We found that S-scores of significantly different distributions were no greater than 0.3 for 94% of significantly different distributions, i.e. 30% or less samples caused the difference. Distributional shape differences were characterized by: a) peak height differences for fibre fractions and b) number of peaks and peak value differences for diffusion direction angles. Larger difference in shape resulted in larger difference in mean values or principle diffusion direction angles. Mean angular differences in principle diffusion directions were 2.751° and 36.776° for significantly different [ϕ_1_,θ_1_] and [ϕ_2_,θ_2_] respectively. Their 95^th^ percentile CAUs were 53.2° and 83.5° respectively. We see the effect of larger CAUs, especially for [ϕ_2_,θ_2_], stemming from angle samples that are antiparallel to each other or interchangeable angle samples between two viable sub-fibre population. We saw from Fig 8 that between CPU and GPU, one may produce coherent PDD (e.g. GPU samples from Fig 8) while the other produce two clusters of PDD that are located antiparallel to each other (e.g. CPU samples from Fig 8). Also, as depicted from Fig 10, two viable sub-fibre populations in a region where crossing-fibres are present can be mislabeled between CPU and GPU and thus create larger angular differences and uncertainties. When swapping those fibres to align for correct sub-fibre population, the differences between PDDs decreased overall. Algorithms such as *probtrackx* does tract streamlining with tract propagation constraints that propagate streamlines smoothly, and avoid internal looping or sharp turns. This is achieved by treating antiparallel angles as the same (i.e. multiplying antiparallel angles by -1 prior to propagation), and sampling from fibre-population that has minimal angular difference from previous propagation direction. These constraints would effectively allow consistent tract streamlines to be produced from CPUs and GPUs, despite the difference in PDF distributional shapes in the PDD angles. Previously, Jbabdi et al. have reported similar inconsistency in sub-fibre population labeling in bedpostx and they consolidated the orientations of sub-fibres by performing a swaping operation, similar to our method of swapping the angular samples where *f*_*2*_>*f*_*1*_ [27] which resulted in better aligned mean directions between CPU and GPU. We note that one of the limitations of this current work is that there was no investigation into effect of using multi-shell models while doing the comparisons. It is reasonable to suggest that with higher b-values there will be better angular resolutions which might lead to better agreement between CPU and GPU results. The challenge in investigating this would be that the CPU *bedpostx* process will take far too long compared to the GPU as much more data need iterative estimation in a linear fashion, and one would require the use of multi-core High Power Computing resources to do this type of investigation in a reasonable amount of time. Still, b = 1000 with a monoexponential model estimation is valuable to investigate between CPU and GPU as most clinical MRI can acquire this type of data with conventional MR system setup and speed-up in *bedpostx* in the GPU can be most effective in this type of front-line clinical setting. Another limitation of this work is that the random-number generator type is not the same between CPU and GPU and thus there is no way of telling how much effect the random number generators have on the differences observed between the two algorithms. The authors have looked at preliminary data where the GPU *bedpostx* algorithm was modified to use the linear-congruential random number generator to obtain the same amount of samples and when compared against the CPU samples, they appeared to have similar amount and magnitude of difference as this current work, which suggests the effect of random number generator in producing differing results would be small. This would then lead us to believe that sample differences are more attributable to difference in implementation of CPU-GPU precision points, math libraries between CPU and GPU-CUDA and more importantly the operation order: L-M initialization then MCMC sequentially v.s. L-M parallel then MCMC parallel. As DWI data are collected with greater amount of gradient directions, combination of different b-values, higher-resolutions, conventional DWI post-processing steps will require more computational resources to finish processing in a reasonable amount of time, and GPUs can offer qualitatively the same results with minimal quantitative difference compared to underlying uncertainty with excellent speed [4]. In summary, although significant differences were found between outputs of CPU and GPU *bedpostx* parameter distributions, differences may have limited impact upon stochastic tractography with single-shell DTI data: a) differences were observed in only 22% of total distributions; b) differences were sparsely distributed in major tract areas; c) differences in fibre orientations were small compared to background angular uncertainty. The latter appears to arise from antiparallel angles and random assignment of principle directions in the presence of multiple viable sub-fibre populations which are not problematic in fibre-tracking using ProbtrackX. It appeared that the combination of differences in operation orders between CPU and GPU and math-library differences affects the magnitude of difference between the two algorithms and collecting data with optimized SNR along with offline post-processing steps to reduce noise level may improve output consistency between the two algorithms.
